# Case of Diabetes Treated with Codeia

**Published:** 1883-10

**Authors:** 


CASE OF DIABETES TREATED WITH CODEIA.

Dr. Bradbury narrated a case of diabetes mellitus, under his care in Addenbrooke's Hospital, benefited by the administration of codeia. The patient was a maltster, aged 69, who came under treatment in September, 1882, with a history that he had had no previous illness of importance, and who had been temperate, robust and remarkably stout. About two and a half years back, he had noticed that he passed an excessive quantity of water ; he then began to suffer from neuralgic pains about the left eyeball, slight dimness of vision, loss of flesh, hunger and thirst. He then came into the hospital for some months, and was treated with salicylic acid. After some improvement was maintained for about twelve months, early in 1882 he began to have a recurrence of the symptoms. In September, 1882, it was reported that vision had been entirely lost in the left eye, and much impaired in the right. Sharp darting pains had occurred in the limbs during the last three months, and had affected the lower limbs with great severity. He had also suffered from giddiness. There were signs of cataract in both eyes, with whitish patches in both retinæ, but no hæmorrhagic patches. The gait was peculiar, as he walked with short strides and some hesitation, and a difficulty in turning round. Sensation in the limbs was normal. Patellar, spermatic, abdominal and posterior scapular reflexes were quite absent. The urine had a specific gravity of 1037 ; it contained a large quantity of sugar, but no albumen. For seven days he was allowed the ordinary hospital diet, and no drug was given; the daily average of urine was 84 ozs., and there was no change in the symptoms. For the next twenty days his diet was restricted to meat, greens, eggs, tea, brown bread, butter, and beef-tea, the daily average of urine being 52 ounces, and slight improvement being noted in the pain in the limbs. During the next seven days half a grain of codeia was given daily, and the daily average of urine was 48 ozs. Then for ten days a grain of codeia was given daily, and the average of urine was reduced to 45 ozs. From November 1 to December 8, the dose of codeia was one grain and a half daily, and the average urine was 40 ozs. During this period the pain was to a great extent lost, and the sight improved in the right eye, and perception of light returned in the left. After December 8, until he left the hospital, he took two grains of codeia daily, and passed on an average 33 ozs. of urine. During the whole of his stay in the hospital, the specific gravity of the urine was scarcely at all affected, varying from 1034 to 1038, and his weight varied from 12 st. 9 lbs. to 12 st. 12 lbs., but was not affected by the treatment. When discharged, in January, 1883, the report was that the man had for some weeks been free from pain; the sight had improved to a slight extent in the right eye; that the absence of patellar reflex continued, and that the gait had not improved, though this latter symptom was attributed to the deficiency in sight. Dr. Bradbury said, in reply to the discussion which followed, that the case was especially interesting, on account of the presence of nervous symptoms, and the marked improvement in them and in the polyuria by the treatment adopted. He had only that day seen another case in consultation in which there was much the same gait, shooting pains in the legs, and a complete absence of the knee-phenomenon. In this case, there was a gouty history; and Dr. Bradbury said he had under his care a gentleman who had had several attacks of gout, had now difficulty of walking, and in whom the patellar reflex was totally abolished. In none of these cases was there any ataxy. A close relationship may exist between gout and glycosuria, and these cases usually ran a somewhat protracted course, quite different from the diabetes occurring in young persons in whom there was no gouty history.—*British Medical Journal.*

